# The diversity of population responses to environmental change

**DOI:** 10.1111/ele.13195

**Published:** 2018-12-09

**Authors:** Fernando Colchero, Owen R. Jones, Dalia A. Conde, David Hodgson, Felix Zajitschek, Benedikt R. Schmidt, Aurelio F. Malo, Susan C. Alberts, Peter H. Becker, Sandra Bouwhuis, Anne M. Bronikowski, Kristel M. De Vleeschouwer, Richard J. Delahay, Stefan Dummermuth, Eduardo Fernández‐Duque, John Frisenvænge, Martin Hesselsøe, Sam Larson, Jean‐François Lemaître, Jennifer McDonald, David A.W. Miller, Colin O'Donnell, Craig Packer, Becky E. Raboy, Chris J. Reading, Erik Wapstra, Henri Weimerskirch, Geoffrey M. While, Annette Baudisch, Thomas Flatt, Tim Coulson, Jean‐Michel Gaillard

**Affiliations:** ^1^ Interdisciplinary Center on Population Dynamics University of Southern Denmark Campusvej 55 5230 Odense M Denmark; ^2^ Department of Mathematics and Computer Science University of Southern Denmark Campusvej 55 5230 Odense M Denmark; ^3^ Institute of Biology University of Southern Denmark Campusvej 55 5230 Odense M Denmark; ^4^ Species360 Conservation Science Alliance 7900 International Drive, Suite 1040 Bloomington MN 55425 USA; ^5^ Centre for Ecology and Conservation College of Life and Environmental Sciences University of Exeter Cornwall Campus, Penryn Cornwall TR10 9EZ UK; ^6^ Evolution and Ecology Research Centre and School of Biological, Earth and Environmental Sciences University of New South Wales Sydney NSW 2052 Australia; ^7^ Department of Evolutionary Biology and Environmental Studies University of Zurich Winterthurerstrasse 190 CH‐8057 Zurich Switzerland; ^8^ Info Fauna Karch UniMail Bâtiment G, Bellevaux 51 2000 Neuchâtel Switzerland; ^9^ Department of Zoology University of Oxford Oxford OX2 6GG UK; ^10^ Departamento de Ciencias de la Vida Universidad de Alcalá 28805 Madrid Spain; ^11^ Departments of Biology and Evolutionary Anthropology Duke University Durham NC 27708 USA; ^12^ Institute of Primate Research National Museums of Kenya Nairobi Kenya; ^13^ Institut of Avian Research An der Vogelwarte 21 D‐26386 Wilhelmshaven Germany; ^14^ Department of Ecology, Evolution, and Organismal Biology Iowa State University 251 Bessey Hall Ames IA USA; ^15^ Centre for Research and Conservation Royal Zoological Society of Antwerp Koningin Astridplein Antwerpen Belgium; ^16^ National Wildlife Management Centre Animal and Plant Health Agency Woodchester Park Nympsfield Gloucestershire GL10 3UJ UK; ^17^ Department of Anthropology Yale University New Haven CT 06511 USA; ^18^ Amphi Consult Sciencepark NOVI, Niels Jernes Vej 10 DK 9220 Aalborg Ø Denmark; ^19^ Department of Anthropology University of Pennsylvania Philadelphia PA USA; ^20^ Université Lyon 1 CNRS UMR 5558 Laboratoire de Biométrie et Biologie Evolutive F‐69622 Villeurbanne France; ^21^ Department of Ecosystem Science and Management Pennsylvania State University 411 Forest Resources Building University Park PA 16802 USA; ^22^ Department of Conservation Te Papa Atawhai PO Box 4715 Christchurch 8140 New Zealand; ^23^ College of Biological Sciences Department of Ecology, Evolution and Behavior University of Minnesota 123 Snyder Hall, 1475 Gortner Ave Saint Paul MN 55108 USA; ^24^ Department of Ecology and Evolutionary Biology University of Toronto 25 Willcocks Street Toronto ON Canada M5S 3B2; ^25^ Centre for Ecology and Hydrology CEH Wallingford Benson Lane, Crowmarsh, Gifford, Wallingford Oxfordshire OX10 8BB UK; ^26^ School of Biological Sciences University of Tasmania Private Bag 5 Hobart TAS Australia; ^27^ Centre d'Etudes Biologiques de Chizé CNRS 79360 Villiers en Bois France; ^28^ Edward Grey Institute Department of Zoology University of Oxford South Parks Road Oxford OX1 3PS UK; ^29^ Department of Public Health University of Southern Denmark Odense 5000 Denmark; ^30^ Department of Biology University of Fribourg Ch. du Musée 10 1700 Fribourg Switzerland

**Keywords:** Age‐structured population models, Bayesian inference, fecundity, mortality, survival

## Abstract

The current extinction and climate change crises pressure us to predict population dynamics with ever‐greater accuracy. Although predictions rest on the well‐advanced theory of age‐structured populations, two key issues remain poorly explored. Specifically, how the age‐dependency in demographic rates and the year‐to‐year interactions between survival and fecundity affect stochastic population growth rates. We use inference, simulations and mathematical derivations to explore how environmental perturbations determine population growth rates for populations with different age‐specific demographic rates and when ages are reduced to stages. We find that stage‐ vs. age‐based models can produce markedly divergent stochastic population growth rates. The differences are most pronounced when there are survival‐fecundity‐trade‐offs, which reduce the variance in the population growth rate. Finally, the expected value and variance of the stochastic growth rates of populations with different age‐specific demographic rates can diverge to the extent that, while some populations may thrive, others will inevitably go extinct.

## Introduction

During the last century, the species extinction rate has increased to more than 1000 times the background rate, and the number of threatened species continues to rise (Barnosky *et al*. 2011; Ceballos *et al*. 2015). Extinction risk is associated with anthropogenic activities and their consequences, with climate change playing a critical role (Pearson *et al*. 2014; Pacifici *et al*. 2015). Climate change can influence extinction risk by increasing temporal variation in demographic rates such as survival and fecundity, which in turn reduces long‐run population growth rates (Pearson *et al*. 2014). However, survival and fecundity are not only affected by environmental conditions. There is abundant evidence that they also change with individual differences in either unmeasured traits such as frailty (Vaupel *et al*. 1979; see review in Gimenez *et al*. 2017) or measured traits such as phenotypic (e.g. Plard *et al*. 2015) or genetic (David 1998) characters. The amount of individual differences within a given population influences its dynamics (Hamel *et al*. 2018). Among traits that shape individual differences, age variation strongly influences demographic rates in response to biological factors such as growth, maturation and senescence (Kirkwood & Austad 2000; Partridge 2010). Thus, to understand population dynamics in variable environments, we must discover how these biological and environmental processes interact to determine demographic rates and population growth.

Species across the tree of life exhibit a wide diversity of age‐specific survival and fecundity patterns (Jones *et al*. 2014). These age‐patterns in demographic rates are often related to a gradual deterioration of physiological functions with age after maturity known as senescence (Jones *et al*. 2008; Nussey *et al*. 2013). This deterioration that is associated with a multitude of genes (Partridge 2010; Olsson *et al*. 2018), results in a monotonic decline in age‐specific survival and fecundity with increasing age after maturity. Moreover, demographic rates change in response to environmental factors such as local weather variables and large‐scale climatic processes (Gaillard *et al*. 2000; Sandvik *et al*. 2008). Factors influencing demographic rates can therefore be divided into two broad categories: (1) a genetic component that dictates the age‐specific schedules of survival and fecundity; and (2) environmental effects that produce departures from these age‐specific demographic trajectories. Although there is increasing interest in unravelling how these mechanisms interact to shape demographic rates and population growth, our current knowledge is still insufficient to make any broad generalisation.

Our understanding of the effect of the environment on age‐structured population dynamics stems primarily from theoretical studies (Tuljapurkar & Orzack 1980; Tuljapurkar 1982a; Coulson *et al*. 2005; Engen *et al*. 2005, 2013; Tuljapurkar & Haridas 2006). Of particular interest are the yearly population growth rates, λ_*t*_, and its expected value, E[λ_*t*_] = λ_*e*_, where E[.] denotes expectation (i.e. the theoretical mean), as well as the logarithm of its geometric mean, E[ln λ_*t*_] = E[*r*_*t*_] = *r*_*e*_, known as the long‐run stochastic population growth rate. Several authors have demonstrated that the long‐run stochastic population growth rate, *r*_*e*_, is always lower than ln[λ_*e*_] as a direct result of Jensen's inequality (Tuljapurkar 1989). Furthermore, the long‐run stochastic population growth rate, *r*_*e*_, and, to a lesser extent the expected value λ_*e*_, often decline with increasing environmental variation (Lewontin & Cohen 1969; Boyce 1977; Tuljapurkar 1982a). However, both Cohen (1979) and Tuljapurkar (1989) noted that when the demographic rates of a long‐lived organism are serially correlated (i.e. demographic rates at time *t* are dependent on their values at time *t* − 1), both population growth rates could sometimes increase with increasing environmental variation. In addition to these average measures, to fully characterise long‐term population dynamics it is fundamental to understand the variance in the yearly population growth rate, Var[λ_*t*_] = *V*_λ_. This variance is determined by the variances and covariances between the age‐specific survival and fecundity rates, which are generated by the environment (Brown *et al*. 1993; Saether & Bakke 2000; Doak *et al*. 2005). Commonly, it is assumed that survival and fecundity have either null or positive covariation (Lee *et al*. 2017), despite increasing evidence of within‐year trade‐offs between survival and fecundity (i.e. negative covariation) in natural populations (Cox *et al*. 2010; Dobson & Jouventin 2010; Robinson *et al*. 2012). Furthermore, either due to data limitation or for illustration purposes, models are often tested on a reduced number of age classes (e.g. two classes, juveniles vs. adults).

Although the theory of population dynamics in stochastic environments is well‐advanced (for a review see Boyce *et al*. 2006), three important questions have received little attention so far. First, given the diversity of age‐specific trajectories of survival and fecundity in the wild, can we expect that all populations will respond similarly to the environment? Second, how much information is lost when estimating stochastic population growth rates and their distributions by reducing age‐specific demographic rates to broad classes? And finally, how does the yearly covariation between survival and fecundity affect population growth rates?

**Figure 1:**
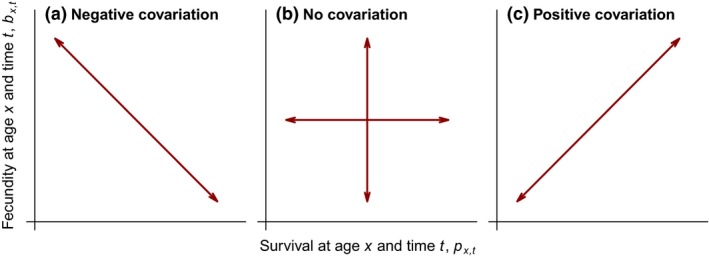
Schematic representation of the three scenarios tested.

To address these questions, we used detailed longitudinal individual‐based data collected from 24 vertebrate species to assess the diversity of age‐trajectories of mortality in wild populations (Table S1). Next, using some of these mortality profiles in combination with a range of age‐specific fecundity trajectories, we employed stochastic simulations, theoretical decompositions of the expected value, λ_*e*_, and variance, *V*_λ_, and approximations to the long‐run stochastic population growth rate, *r*_*e*_. We used these simulations and decompositions to compare the dynamics of populations with different age‐specific demographic rates, and to determine the performance of models with a reduced number of age classes, as commonly used in management and conservation studies. We explored three scenarios: (a) within‐year trade‐offs between survival and fecundity (i.e. negative covariation); (b) no covariation between survival and fecundity (i.e. they vary independently); and (c) positive covariation between these demographic rates (Fig. 1). Finally, we determined the relationship between the age‐specific trajectories, the magnitude of the environmental variation and the average time to extinction.

## Methods

### Mortality trajectories in the wild

We obtained 24 long‐term individual‐based data sets from wild vertebrate populations (mammals, birds, reptiles and amphibians) that covered a range of positions along the slow‐fast life history continuum (Table S1) (Gaillard *et al*. 1989, 2016). The data sets were either census data with almost complete detection or typical capture‐mark‐recapture/‐recovery (CMRR) data. We combined data for males and females since sex information was unavailable for several data sets. For inference on age‐specific mortality we used the R package BaSTA (Colchero & Clark 2012; Colchero *et al*. 2012). We tested 10 different functional forms of age‐specific mortality, including two and three parameter Weibull, Gompertz and Gompertz‐Makeham, Logistic, and combinations of these with an initial declining juvenile mortality and, finally, a model with a single adult stage (i.e. constant adult mortality). We quantified the support for each model based on the deviance information criterion (DIC) (Spiegelhalter *et al*. 2002; Celeux *et al*. 2006).

### Stochastic simulation modelling

We simulated five age‐specific mortality trajectories based on the results above, alongside five simulated fecundity trajectories to reflect a wide range of life histories (upper left panel in Fig. 3). For each combination of mortality and fecundity, we constructed fully age‐dependent deterministic Leslie matrices (**A**_*a*_) as well as the corresponding deterministic matrices with constant adult survival and fecundity (**A**_*c*_), both with stationary deterministic population growth rates (i.e. λ_*d*_ = 1). We ran 2000 short‐term stochastic simulations of 200 time steps each, for every combination of survival and fecundity, where we randomly perturbed the demographic rates in both matrices through time. We calculated average population growth rates, ${\overset{¯}{\lambda }}_{a}$ and ${\overset{¯}{\lambda }}_{c}$, and their densities, and quantified the amount of information lost if we approximated the density of ${\overset{¯}{\lambda }}_{a}$ with the density of ${\overset{¯}{\lambda }}_{c}$ by means of the Kullback–Leibler information (Kullback & Leibler 1951).

We tested three scenarios, namely (a) where environmental shocks affected survival in the opposite direction to fecundity (i.e. negative covariation), as expected with year‐to‐year survival‐reproduction trade‐offs; (b) where there was no covariation between demographic rates, and (c) where survival and fecundity varied in the same direction and magnitude (i.e. positive covariation), as generally performed in case study analyses (Fig. 1; for further details see Supporting Information).

### Decomposition of E[λ_*t*_] = λ_*e*_ and Var[λ_*t*_] = *V*_λ_

The yearly population growth rate can be calculated as

(1) $$\lambda_{t}=\sum_{x=0}^{\omega }w_{x,t-1}\left(b_{x,t}+p_{x,t}\right),\;\text{for}\;t=0,1,2,\ldots$$

where *x *=* *0, 1, 2, …, ω are ages, *w*_*x,t*−1_ is the proportion of individuals of age *x* at time *t* − 1, and *b*_*x*,*t*_ and *p*_*x*,*t*_ are the age‐specific fecundity rates and survival probabilities at time *t*, respectively (Tuljapurkar 1990). From eqn (1), we used moment estimation and structured demographic accounting (Brown & Alexander 1991; Brown *et al*. 1993) to derive theoretical decompositions of the expected value of the yearly population growth rate, E[λ_*t*_] = λ_*e*_, and its variance, Var[λ_*t*_] = *V*_λ_ (see full derivations in Supporting Information). In order to explore our derivation on the full‐age‐dependent and the one‐adult‐stage models, we ran a single simulation of 10 000 time steps for each of the 25 combinations of age‐specific fecundity and survival, with which we confirmed that our decompositions were exact.

### Approximations to E[*r*_*t*_] = *r*_*e*_

The long‐run population growth rate is given by the logarithm of the geometric mean of λ_*t*_, this is E[ln λ_*t*_] = *r*_*e*_. Today, *r*_*e*_ is often estimated by means of the small noise approximation provided by Tuljapurkar (1982b), given by

(2) $$r_{e}\approx \ln\lambda_{0}-\frac{\tau_{0}}{2\lambda_{0}^{2}},$$

where λ_0_ is the dominant eigenvalue of the matrix of average demographic rates and τ_0_ accounts for the covariances between these demographic rates scaled by the sensitivities of λ_0_ to them (i.e. a measure of the variance in λ_*t*_). It is important to note that λ_0_ is a theoretical quantity that requires calculating the average demographic rates that result from the full variation in the environmental sequence, and thus it may not always be directly equivalent to the asymptotic population growth rate, λ_*d*_, derived from the deterministic matrix calculated from short‐term average demographic rates. Here, we use the results in the previous section to calculate a second order Taylor approximation of *r*_*e*_ based on λ_*e*_ and *V*_λ_ (see Supporting Information) and used simulations to determine their accuracy.

### Mean time to extinction

For each combination of survival and fecundity and each scenario, we simulated 500 populations for 2000 time steps accounting not only for environmental stochasticity but also for demographic stochasticity (Engen *et al*. 2005) (see Supporting Information). We extracted the population sizes at the end of each simulation and calculated the average time to extinction, defined as the average time each population reaches a population size under one individual.

## Results

### Mortality trajectories in the wild

**Figure 2:**
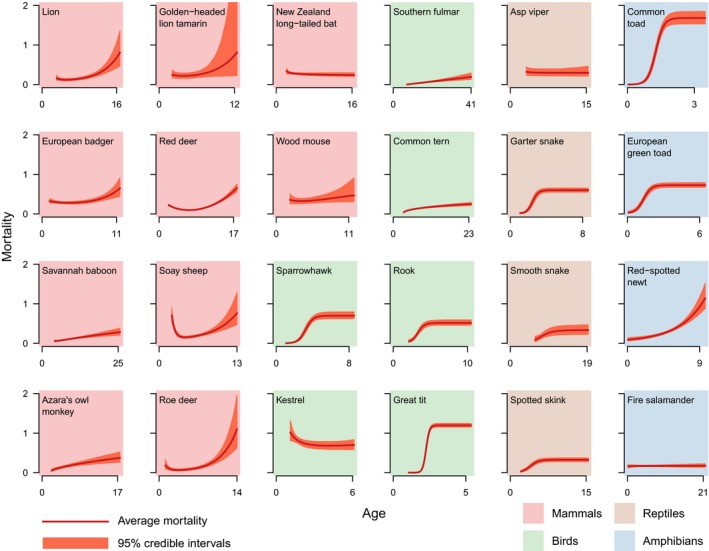
Best‐fitting models of age‐specific mortality during adulthood for 24 species of terrestrial vertebrates compared to models including only age‐independent adult mortality. Age units are in years, except for the wood mouse, where it is in months. Background color indicates the taxonomic class. Age zero for all amphibians indicates the time when they transitioned from the post‐metamorphic juvenile stage to the adult stage.

Our analysis of individual‐based data on 24 wild species (Fig. 2) showed that adult mortality changed with age, with no consistently favoured model. A bathtub‐shaped mortality trajectory (Siler 1979) was the most commonly supported model in ungulates and mammalian carnivores. We found clear monotonic increases in mortality from maturity onwards in two primates [savannah baboon (*Papio cynocephalus*) and Azara's owl monkey (*Aotus azarae*)] and two seabirds [common tern (*Sterna hirundo*) and southern fulmar (*Fulmarus glacialoides*)]. The first three of these were best characterised by a decelerating Weibull function (Pinder *et al*. 1978). In addition, we found logistic mortality curves in birds, reptiles and amphibians (Pletcher 1999; Vaupel & Missov 2014). Surprisingly, for the New Zealand long‐tailed bats (*Chalinolobus tuberculatus*), we found that the best model was a declining three‐parameter Weibull model.

### Stochastic simulation models

**Figure 3:**
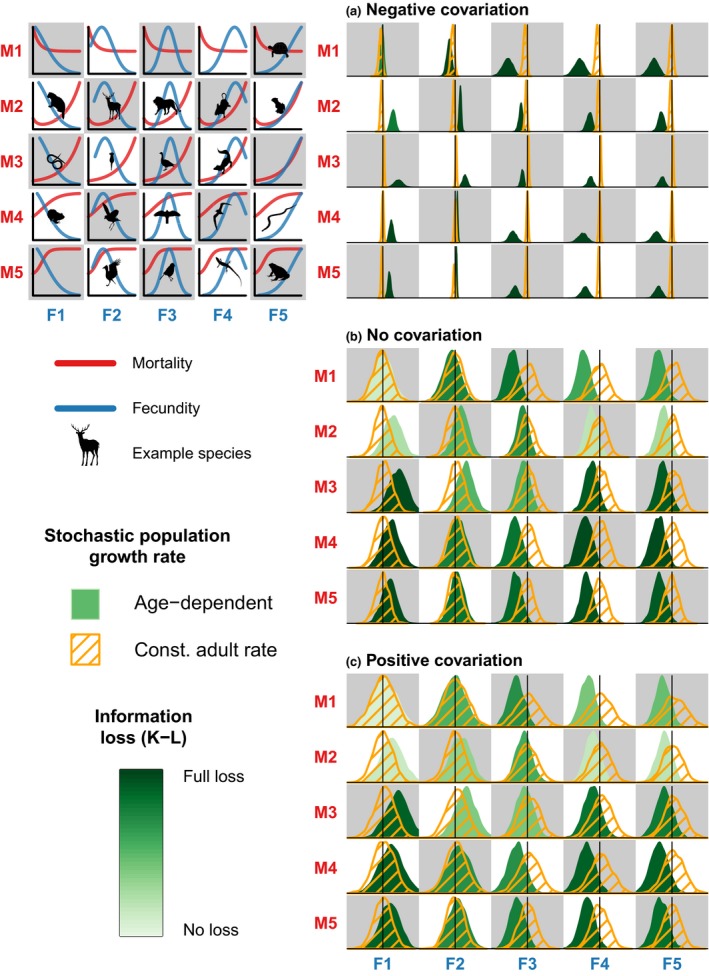
Densities of the average population growth rates ${\overset{¯}{\lambda }}_{a}$ and ${\overset{¯}{\lambda }}_{c}$ derived by using the fully age‐dependent and the one‐adult‐stage models, respectively. We modelled three scenarios: (a) negative yearly covariation between survival and fecundity (i.e. a trade‐off) as a function of environmental shocks; (b) no covariation between demographic rates; and (c) positive covariation between survival and fecundity. The tones of green on the density of ${\overset{¯}{\lambda }}_{a}$ correspond to the level of Kullback–Leibler (K–L) information loss when predicting the density of ${\overset{¯}{\lambda }}_{a}$ with ${\overset{¯}{\lambda }}_{c}$. The first panel on the left shows the 25 combinations of age‐specific mortality and fecundity tested. The silhouettes in each panel indicate species for which the trends in mortality and fecundity can roughly be described by the trajectories in the matching plot. These are only for reference purposes and are not intended as an accurate depiction of the species’ demographic rates. The checker box format (white and grey squares) with codes M1‐M5 (for mortality) and F1‐F5 (for fecundity) is meant to facilitate matching the combination of demographic rates with the corresponding results plot.

We found that the short‐term arithmetic mean of the population growth rate, $\overset{¯}{\lambda }$, can increase in response to environmental stochasticity (Fig. 3). This is particularly likely for life‐histories with senescent or bathtub‐shaped mortalities and with reproductive senescence or hump‐shaped fecundity with early onset of senescence, as observed in most mammals and birds. Furthermore, we found large differences in the distribution of $\overset{¯}{\lambda }$ between the one‐adult‐stage and the fully age‐dependent models under the scenario with trade‐offs between survival and fecundity (i.e. negative covariation) and when these demographic rates varied independently (i.e. no covariation). The differences were moderate to low in the scenario where survival and fecundity covaried positively. Importantly, in all cases, the models with constant adult survival predicted that populations declined slowly even if their age‐dependent counterparts predicted steep declines in population growth. This was particularly evident for life histories with a late onset of reproductive senescence or with increasing fecundity (i.e. negative reproductive senescence).

### Decomposition of E[λ_*t*_] and Var[λ_*t*_]

We show that for any stochastic population model with transitions given by a Leslie matrix (Leslie 1945), the expected value of the yearly population growth rate is given by

(3) $$\lambda_{e}=E\left[\lambda_{t}\right]=\tilde{\lambda }+C_{\mathrm{wb}}+C_{\mathrm{wp}}\;\text{for}\;t\geq 0,$$

where $\tilde{\lambda }$ is the population growth rate calculated as in eqn (1) but replacing *p*_*x*,*t*_, *b*_*x*,*t*_ and *w*_*x*,*t*−1_ with the average survival probabilities *E*[*p*_*x*,*t*_] = ρ_*x*_, average fecundities *E*[*b*_*x*,*t*_] = β_*x*_, and average age distribution *E*[*w*_*x*,*t*−1_] = η_*x*_, while *C*_*wb*_ and *C*_*wp*_ are the sums across ages of the covariances between *w*_*x*,*t*−1_ and the demographic rates *b*_*x*,*t*_ and *p*_*x*,*t*_, respectively. However, for serially uncorrelated environments, we have

$$C_{\mathrm{wb}}=C_{\mathrm{wp}}=0,$$

thus eqn (3) simplifies to $\lambda_{e}=\tilde{\lambda }$.

**Figure 4:**
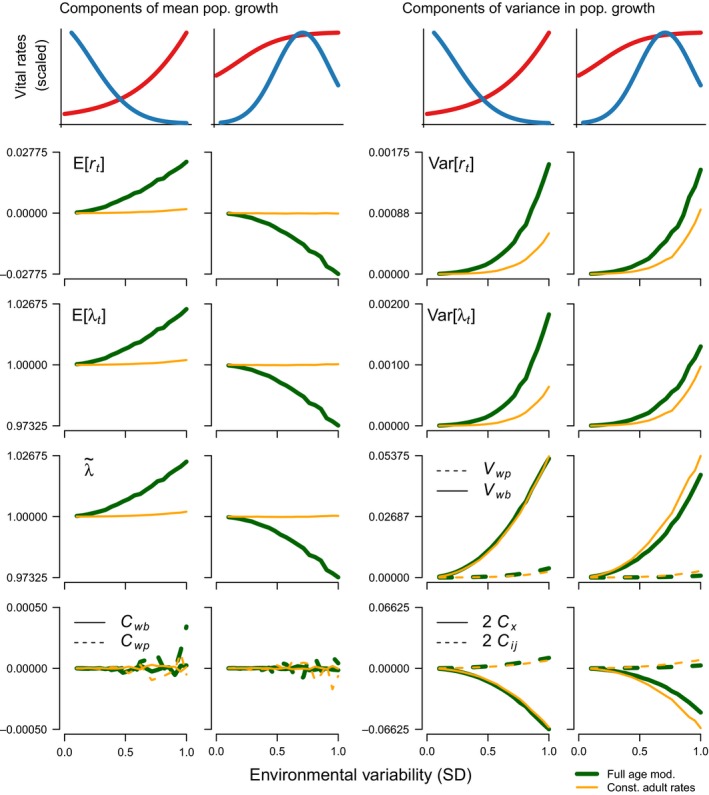
Relationship between environmental variation (measured as the standard deviation of environmental shocks) and the components of E[λ_*t*_] = λ_*e*_ and Var[λ_*t*_] = *V*_λ_ for two combinations of mortality and fecundity profiles (F1‐M3 and F4‐M4 in Fig. 3) under the negative covariation scenario (i.e. trade‐offs between survival and fecundity). The dark green thick lines correspond to the fully age‐dependent models and the orange thin lines to the model with a single adult‐stage.

We found that, depending on the combination of age‐specific mortality and fecundity, both, *r*_*e*_ and λ_*e*_ can increase as the environmental variance increases, most noticeably for the negative covariation scenario (Fig. 4). The increase in *r*_*e*_ is less evident as we move from the negative to the positive covariation scenarios (Figs S1 and S2). This increase in λ_*e*_ is primarily driven by an increase in the population growth rate calculated from the average age‐structure and average demographic rates, $\tilde{\lambda }$, possibly due to marked differences between the average age‐structure and the stable age‐structure of the deterministic matrix (Fig. S3) (Tuljapurkar 1990).

We also show that the variance in the stochastic population growth rate is given by

(4) $$V_{\lambda }=\text{Var}\left[\lambda_{t}\right]=V_{\mathrm{wp}}+V_{\mathrm{wb}}+2C_{x}+2C_{\mathrm{ij}},\;\text{for}\;x,i,j=0,1,\ldots ,\omega \;\text{and}\;i\neq j,$$

where *V*_*wp*_ and *V*_*wb*_ are the sums across ages of the variances in the products *w*_*x*,*t*−1_ *p*_*x*,*t*_ and *w*_*x*,*t*−1_ *b*_*x*,*t*_, respectively, *C*_*x*_ is the sum across ages of the covariances between the products *w*_*x*,*t*−1_ *p*_*x*,*t*_ and *w*_*x*,*t*−1_ *b*_*x*,*t*_, and *C*_*ij*_ is the sum of the cross‐covariances between different ages or stages *i* and *j* (Fig. 4). This result is consistent with the derivation proposed by Brown *et al*. (1993).

**Figure 5:**
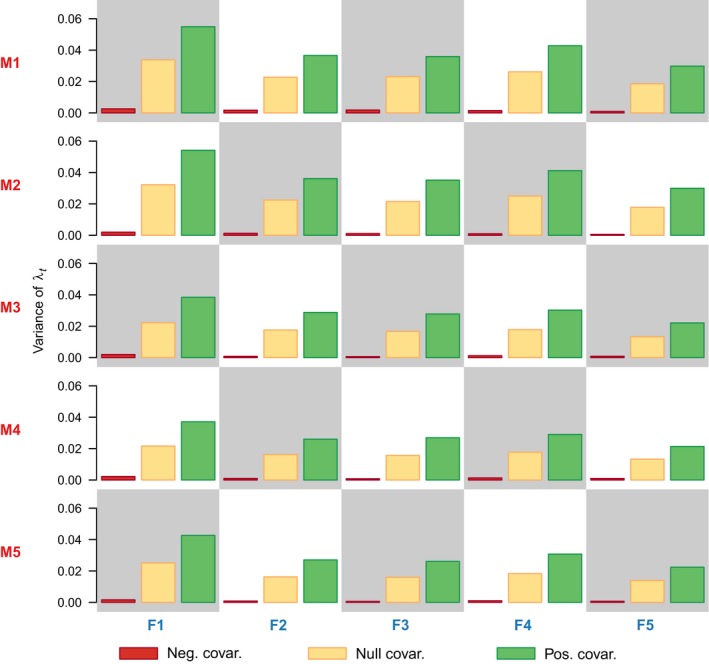
Magnitude of the variance of λ_*t*_ under the three different scenarios (i.e. negative covariation, null covariation, and positive covariation), for all combinations of demographic rates. The combinations of mortality (M1‐M5) and of fecundity (F1‐F5) are distributed as in Fig. 3.

Based on our simulations, we find that the variance in the stochastic population growth rate scales over the three different scenarios as

$$V_{\lambda^{-}}<V_{\lambda 0}<V_{\lambda^{+}},$$

where the subscripts ‘−’, ‘0’, and ‘+’, refer to scenarios of negative, null, and positive covariation between survival and fecundity, respectively, for all combinations of survival and fecundity (Fig. 5). In other words, in the presence of year‐to‐year trade‐offs between survival and fecundity (i.e. negative covariation), the variance in the stochastic population growth rate is lowest and increases as the covariation changes from negative to positive. Furthermore, for the scenario with negative covariation between fecundity and survival, we find that the components of *V*_λ_ with the largest magnitude are *V*_*wp*_ and *C*_*ij*_ although the latter is commonly negative, which reduces the overall variance (Fig. 4). As our scenarios transition from negative covariation to null or positive covariation, the magnitude of the cross‐covariances *C*_*ij*_ decreases considerably, which in turn increases *V*_λ_ (Figs S1 and S2). These results are consistent with proofs from Tuljapurkar (1982a), and later derivations and applications from Brown *et al*. (1993) and Doak *et al*. (2005) that show that within year negative covariation between demographic rates reduce the variance in the population growth rate.

### Approximation for E[*r*_*t*_] = *r*_*e*_

We provide the second‐order Taylor approximation of *r*_*e*_ given by

(5) $$r_{e}\approx r_{T}=\ln\left(\lambda_{e}\right)-\frac{1}{2\lambda_{e}^{2}}V_{\lambda }$$

and therefore λ_*s*_ ≈ λ_*T*_ = exp(*r*_*T*_) (see Supporting Information). The approximation in eqn [Link] is close to the small noise approximation proposed by Tuljapurkar (1982b) in eqn (2). However, in his full approximation, Tuljapurkar included a third term that accounts for serial correlation in the environmental sequence. As we mention above, the effect of serial correlation is incorporated in the calculation of λ_*e*_ from eqn (3) through the *C*_*wb*_ and *C*_*wp*_ terms. We show that our approximation is generally very close to the empirical value of λ_*s*_ calculated from long‐term simulations (i.e. *t *=* *100 000 with burn‐in = 1000) (Fig. S4).

### Mean time to extinction

**Figure 6:**
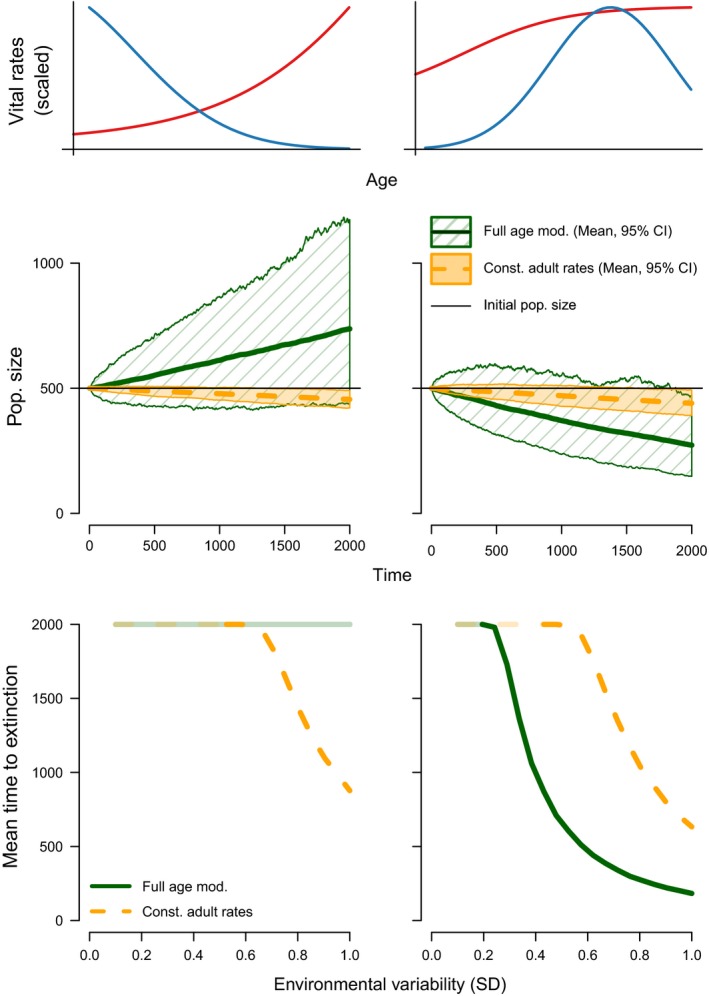
Population sizes after 2000 time steps and mean time to extinction for two combinations of mortality and fecundity (F1‐M3 and F4‐M4 in Fig. 3) under the negative covariation scenario (i.e. trade‐offs between survival and fecundity). The lightly shaded lines in the lower panels indicate that no populations went extinct.

Our models show that mean time to extinction depends strongly on life history and covariation among demographic rates. After 2000 time steps, populations for which λ_*e*_ increases with increasing environmental variation may never go extinct, particularly under the negative covariation scenario (Fig. 6). When there is no covariation between demographic rates (scenario b) a fraction of the populations go extinct only with large environmental variation (Fig. S5). Under positive covariation in demographic rates, populations start going extinct at moderate values of environmental variation (Fig. S6). Noticeably, models in which adults are pooled into a single age class, average population sizes always decline with increasing environmental variation, irrespective of the behaviour of their fully age‐dependent counterpart.

## Discussion

Understanding and predicting the dynamics of populations in their natural environment is becoming ever more urgent due to the dramatic increase in the number of species threatened with extinction and the looming threat of more variable and unpredictable environments (Pearson *et al*. 2014; Pacifici *et al*. 2015; Palmer *et al*. 2017). Our results contribute to unifying the well‐developed fields of ageing research and age‐structured population dynamics by providing unequivocal evidence of the diversity of age‐specific demographic rates in nature and showing that populations with these diverse demographic rates respond to variable environments in markedly different ways.

Our analysis of longitudinal data from 24 vertebrate populations supports recent empirical results suggesting a greater diversity of age‐specific demographic trajectories in natural populations than previously thought (Jones *et al*. 2014). We find bathtub shaped mortalities and mortalities increasing as a power function of age [i.e. Weibull function (Pinder *et al*. 1978)] in mammals, Weibull and logistic mortalities in birds, reptiles and amphibians, and few populations of reptiles and amphibians with exponentially increasing mortality with age [i.e. Gompertz function (Gompertz 1825)]. It is important to note that the apparent deceleration in mortality with increasing age in the two primate species results from pooling males and females in the models, which results in demographic heterogeneity (Vaupel & Yashin 1985; Aalen 1994). Notwithstanding these caveats, our results show that the assumption of constant adult mortality in these populations is never appropriate.

Since the pioneering work of Eberhardt (1985), who first warned against neglecting age structure and in particular senescence when assessing population dynamics, several studies have suggested that measures of population performance may be strongly affected by the age‐trajectories of mortality and fecundity (Gaillard *et al*. 2000; Delgiudice *et al*. 2006; Salguero Gómez & Plotkin 2010; Schindler *et al*. 2012; Sæther *et al*. 2013). Robert *et al*. (2015) found that senescence accelerated the extinction risk of mammal populations. Although their study provided an important starting point for understanding the relationship between senescence and extinction risk, they failed to account for age‐dependence in fecundity. Here, by exploring a large diversity of age‐specific demographic rates, we showed that: (1) age‐structured population models that aggregate age‐classes into broad stages (e.g. juvenile and adult), invariably showed reduced variance in the yearly population growth rate (i.e. Var[λ_*t*_] = *V*_λ_) and declining arithmetic and geometric mean population growth rates (i.e. λ_*e*_ and λ_*s*_) with increasing environmental variation, even when these rates increased for their fully age‐dependent counterparts (Figs 3 and 4); (2) λ_*e*_ and λ_*s*_ often decreased, as commonly assumed, but could also increase depending on the age‐trajectories of survival and fecundity (Fig. 4; Figs S1, S2, and S4); and (3) survival‐fecundity trade‐offs reduced the variance of the yearly population growth rate (Fig. 5), thereby dramatically reducing extinction probability with increasing environmental variation (Fig. 6).

Theoretical work predicts that the long‐run stochastic population growth rate, *r*_*e*_ = ln λ_*s*_, should generally decline as the environmental variation increases, even if the expected value E[λ_*t*_] = λ_*e*_ increases (Lewontin & Cohen 1969; Boyce 1977; Tuljapurkar 1982a, 1990). Here, we showed that in some cases both λ_*e*_ and λ_*s*_ can increase with increasing environmental variation, particularly for life‐histories with senescent or bathtub mortality and reproductive senescence (Fig. 3 and Fig. S4). These effects were first observed by Cohen (1979) and later confirmed by Tuljapurkar (1989) for cases where demographic rates were serially correlated among consecutive years. We find that, in the absence of serial correlations, the combination of particular age‐specific demographic rates and their within‐year covariation can also produce this increase in λ_*s*_ and λ_*e*_. In these cases, the long‐term average age structure deviates from the stable age structure of the deterministic matrix (Fig. S3). Tuljapurkar (1990) showed that this departure from the deterministic age‐structure is driven by the covariation between demographic rates. This is particularly noticeable from our results when the variance in the yearly population growth rate, *V*_λ_, is reduced due to positive covariation between survival and fecundity (Fig. 5). Interestingly, not all combinations of age‐specific survival and fecundity produce the same departure from the deterministic age‐structure, which suggests that the covariation between survival and fecundity does not affect equally all combinations of age‐specific demographic rates. Concurrently, Doak *et al*. (2005) stressed the importance of accounting for the covariation between demographic rates in the estimation of stochastic population growth rates. However, applied and theoretical models often assume that survival and fecundity are either independent or positively related (Boyce 1977; Tuljapurkar & Orzack 1980). For example, Lee *et al*. (2017) found that time to extinction for a simulated moose population was not greatly affected by positive covariation between survival and fecundity, compared to models that assumed these rates varied independently. We show here that estimates of λ_*e*_ and λ_*s*_ under null and positive covariation are closer to each other than to the negative covariation scenario (i.e. survival‐fecundity trade‐offs), which is likely to occur in the presence of density‐dependence and therefore in populations close to carrying capacity.

It is worth mentioning that our derivations and simulations did not consider several processes that are known to play an important role in the regulation of demographic rates and stochastic population growth rates. Notably, we did not take into account the effect of density dependence on survival and fecundity (Coulson *et al*. 2001; Lande *et al*. 2002, 2006; Coulson *et al*. 2008; see Bonenfant *et al*. 2009 for a review in large herbivores). Lande *et al*. (2006) showed that the strength of density dependence can be calculated as the sum of the elasticities of the population growth rate at equilibrium to the number of individuals in each age class, which stresses the fundamental role of each age's contribution to the regulation of the population. Also, demographic buffering, defined as a reduction in the sensitivity of key demographic rates to environmental perturbations (Pfister 1998; Boyce *et al*. 2006), can reduce the variance in the stochastic population growth rate (Gaillard *et al*. 2000; Gaillard & Yoccoz 2003; Morris & Doak 2004; Koons *et al*. 2009; Morris *et al*. 2011). Further work to explore how these processes affect the dynamics of populations with different age‐specific demographic rates will provide fundamental insights to the large body of theoretical and applied research on age‐structure population dynamics, while opening new and interesting research opportunities with far reaching consequences for both theoretical and applied population biology.

Wild populations around the globe are becoming increasingly vulnerable to extinction due to anthropogenic activities (Barnosky *et al*. 2011; Ceballos *et al*. 2015), exacerbated by increasing variation in environmental conditions associated with climate change (Pearson *et al*. 2014; Pacifici *et al*. 2015). Further research is needed to deepen our understanding of population dynamics in the wild, particularly in the case of non‐stationary (e.g. increasing average temperatures) and increasingly variable environments as we are witnessing under climate change (IPCC 2012). Our current biodiversity crisis and the looming threat of climate change make these efforts more pressing than ever.

## Authorship

FC and ORJ conceived the project; FC, ORJ and DAC initiated the research and wrote the manuscript, FC implemented the analyses and mathematical derivations, J‐MG, TC and DH provided discussion and insights into the stochastic matrix analysis. TF, FZ and AB provided insights and discussion on the theories of senescence. DH, BRS, AFM, SCA, PHB, SB, AMB, KMD, RJD, SD, EF, TF, JF, MH, SL, J‐FL, JM, DAM, CO, CP, BER, CJR, EW, HW, GMW, J‐MG and TC contributed long‐term data sets and insights into the corresponding species. All co‐authors contributed discussions and edits to the manuscript.

## Data Accessibility Statement

Data available from the Dryad Digital Repository: https://doi.org/10.5061/dryad.d5f54s7.
