# Supplementary material for: The diversity of population responses to environmental change
Source: Ecol Lett. 2018 Dec 9;22(2):342–53. doi: 10.1111/ele.13195 (PMC6378614; doi:10.1111/ele.13195)
Supplement: Supplementary file 1 [file ELE-22-342-s001.pdf]

Supporting Information

The diversity of population responses to environmental change

Fernando Colchero, Owen R. Jones, Dalia A. Conde, David Hodgson, Felix Zajitschek, Benedikt R. Schmidt, Aurelio F. Malo, Peter H. Becker, Sandra Bouwhuis, Anne M. Bronikowski, Kristel M. De Vleeschouwer, Richard J. Delahay, Stefan Dummermuth, Eduardo Fernández-Duque, Thomas Flatt, John Frisenvænge, Martin Hesselsøe, Sam Larson, Jean-François Lemaître, Jennifer McDonald, David A.W. Miller, Colin O'Donnell, Craig Packer, Becky E. Raboy, Chris J. Reading, Erik Wapstra, Henry Weimerskirch, Geoff M. While, Annette Baudisch, Tim Coulson, Jean-Michel Gaillard

Contents

1 Growth rates for populations with same deterministic λ and average adult rates but different number of age classes 1
A Simulation of mortality and fecundity trajectories . . . 1
B Stochastic perturbations to demographic rates . . . 3
C Comparing the distributions of $\bar{\lambda}_a$ and $\bar{\lambda}_c$ . . . 3

2 Review and alternative decomposition of $\mathbf{E}[\lambda_t] = \lambda_e$ and $\mathbf{Var}[\lambda_t] = V_\lambda$ 4
A Expressing λ_t in terms of demographic rates and age structure . . . 4
B Expected value of λ_t . . . 5
C Variance of λ_t . . . 6

3 Approximations to $\mathbf{E}[r_t] = r_e$ 8

1. Growth rates for populations with same deterministic λ and average adult rates but different number of age classes

A. Simulation of mortality and fecundity trajectories. We constructed five mortality rates and five fecundity rates that covered 25 combinations of age-specific survival and fecundity trajectories depicted in the left panel of Fig. 3 in the main text.

We calculated the range of survival trajectories by varying the mortality or hazard rate function, defined as

$$\mu(x|\boldsymbol{\theta}) = \lim_{\Delta x \to 0} \frac{\Pr(x \leq X < x + \Delta x \mid X \geq x, \boldsymbol{\theta})}{\Delta x}, \quad \textbf{[S1a]}$$

where x is age and X is a random variable for ages at death, while $\boldsymbol{\theta}$ is a vector of parameters. From Eq. **S1a**, several demographic functions are derived, namely

$$S(x|\boldsymbol{\theta}) = \Pr(X > x) = \exp\left[-\int_0^x \mu(y|\boldsymbol{\theta})dy\right], \quad \textbf{[S1b]}$$

$$F(x|\boldsymbol{\theta}) = \Pr(X < x) = 1 - S(x|\boldsymbol{\theta}), \quad \textbf{[S1c]}$$

$$f(x|\boldsymbol{\theta}) = \Pr(x < X < x + dx) = \mu(x|\boldsymbol{\theta})S(x|\boldsymbol{\theta}), \quad \textbf{[S1d]}$$

where Eq. **S1b** is the survival probability, Eq. **S1c** is the the probability that death occurs before age x (or cumulative density function, CDF), and Eq. **S1d** is the probability density function (PDF) of ages at death.

We used five baseline age-specific mortality functions where the first (M1) produced a declining mortality with age (47) given by

$$\mu(x|\theta) = \exp(a_0 - a_1 x) + c,$$

where $a_0 \in \mathbb{R}$ and $a_1, c > 0$. The second (M2) produced a bathtub or Siler mortality profile (39) as

$$\mu(x|\theta) = \exp(a_0 - a_1 x) + c + \exp(b_0 + b_1 x),$$

where $a_0, b_0 \in \mathbb{R}$ and $a_1, b_1, c > 0$. The third (M3) produced typical senescent profile with exponentially increasing mortality given by the Gompertz-Makeham model (18, 24) given by

$$\mu(x|\theta) = c + \exp(b_0 + b_1 x),$$

where $b_0 \in \mathbb{R}$ and $b_1, c > 0$. The forth (M4) and fifth (M5) mortality models were constructed using a logistic mortality model (31) of the form

$$\mu(x|\theta) = c + \frac{\exp(b_0 - b_1 x)}{1 + b_2 \frac{e^{b_0}}{b_1}(e^{b_1 x} - 1)},$$

where $b_0 \in \mathbb{R}$ and $b_1, b_2, c > 0$. The main difference between M4 and M5 was given by the intensity of the b_2 parameter, where for M4 b_2 was lowest, producing a decelerating mortality instead of a typical logistic mortality.

From the fully age-structured mortality rates, we calculated survival probabilities at every age interval $[x, x + \Delta x)$ as

$$\begin{aligned} p_x &= \Pr\left(X > x + \Delta x | X > x\right) \\ &= \exp\left[-\int_x^{x+\Delta x} \mu(t)dt\right] \\ &= \frac{S(x + \Delta x)}{S(x)}, \end{aligned} \tag{S2}$$

where $\Delta x = 1$. We modelled age-specific fecundity rate $b(x)$ using the following flexible exponential function with a quadratic effect as a function of age

$$b(x|\boldsymbol{\gamma}) = \gamma_0 e^{-\gamma_1 (x - \gamma_2)^2}, \tag{S3}$$

where $\gamma_0, \gamma_1, \gamma_2 > 0$. In Eq. **S3**, $\boldsymbol{\gamma}$ is a vector of parameters such that γ_0 controls the maximum offspring produced when $x = \gamma_2$, γ_1 determines how fast fecundity rate increases with age, and γ_2 represents the age at which the maximum fecundity is achieved. As with mortality, we varied the values of $\boldsymbol{\gamma}$ to simulate a range fecundity trajectories. We calculated fecundity for a given age interval $[x, x + \Delta x)$ as

$$b_x = \int_x^{x+\Delta x} b(t|\boldsymbol{\gamma})dt. \tag{S4}$$

With the resulting fecundity and survival probabilities, we constructed Leslie matrices (23) for these fully age-structured ($\boldsymbol{A}_a$) trajectories and calculated the intrinsic population growth rate λ as the dominant eigenvalue together with the corresponding stable age distribution as the right eigenvector for λ, $\boldsymbol{u}^\top = [u_0, u_1, \ldots, u_\omega]$ where we define ω as the age when only 0.1% of the population remains alive (i.e. $S(x) = 0.001$) (8).

We then constructed the corresponding two-stage models by assuming that juvenile survival, $p_0, p_1, \ldots, p_{\alpha-1}$, was equivalent for both models with α representing the age at maturity, and calculated adult survival (p_c) and fecundity (m_c) for the two-stage model as the average survival and fecundity for adults ($x \geq \alpha$) in the fully age-structured model as

$$p_c = \mathrm{E}\left[p_{\{x:x\geq\alpha\}}\right] \approx \frac{\sum_{x=\alpha}^{\omega} p_x u_x}{\sum_{x=\alpha}^{\omega} u_x} \tag{S5}$$

and

$$b_c = \mathrm{E}\left[m_{\{x:x\geq\alpha\}}\right] \approx \frac{\sum_{x=\alpha}^{\omega} b_x u_x}{\sum_{x=\alpha}^{\omega} u_x}, \tag{S6}$$

respectively. The resulting Leslie matrices (23) for the fully age-structured ($\boldsymbol{A}_a$) and for the one-adult-stage ($\boldsymbol{A}_c$) schedules are

$$\boldsymbol{A}_a = \begin{bmatrix} 0 & \ldots & b_\alpha & \ldots & b_{\omega-1} & b_\omega \\ p_0 & \ldots & 0 & \ldots & 0 & 0 \\ \vdots & \ddots & \vdots & \ddots & \vdots & \vdots \\ 0 & \ldots & p_\alpha & \ldots & 0 & 0 \\ \vdots & \vdots & \vdots & \ddots & \vdots & \vdots \\ 0 & \ldots & 0 & \ldots & p_{\omega-1} & p_\omega \end{bmatrix} \tag{S7}$$

and

$$\boldsymbol{A}_c = \begin{bmatrix} 0 & \dots & b_c \\ p_0 & \dots & 0 \\ \vdots & \ddots & \vdots \\ 0 & \dots & p_c \end{bmatrix}, \qquad \text{[S8]}$$

respectively, where the deterministic transition matrices produce the same dominant eigen value, this is $\lambda_a = \lambda_c$. We calculated deterministic population growth rates, λ_a and λ_c (8), with function `eigen()` from the `base` package in the statistical software R (34).

B. Stochastic perturbations to demographic rates. To simulate stochastic perturbations for both matrices $\boldsymbol{A}_a$ and $\boldsymbol{A}_c$, we simulated environmental shocks by randomly drawing values z_t and v_t from a normal distribution with mean 0 and variance 1. We then used a logistic function to perturb the age-specific survivals as

$$p_{x,t} = \frac{1}{1 + \exp\left[-g(p_x) - z_t\right]}, \qquad \text{[S9]}$$

where $g(p_x) = \log[p_x/(1 - p_x)]$, which limits $p_{x,t} \in [0, 1]$. Similarly, we used a logistic function for the shocks on fecundity as

$$b_{x,t} = \frac{M}{1 + \exp\left[-h(b_x) - v_t\right]}, \quad \text{for } x \geq \alpha, \qquad \text{[S10]}$$

where $h(b_x) = \log[b_x/(M - b_x)]$ and $M = 2$ is the maximum offspring, which limits the range of values for $b_{x,t} \in [0, M]$.

We simulated three different scenarios: a) negative covariation between survival and fecundity (i.e. $z_t = -v_t$); b) no covariation between survival and fecundity (i.e. z_t and v_t independent); and c) positive covariation between survival and fecundity (i.e. $z_t = v_t$).

For the analysis of extinction times, we included demographic stochasticity following (15) as additional random shocks h_t with mean 0 and variance inversely proportional to the population size, namely $\text{Var}\left[h_t\right] = \sigma_h^2/N_t$, where we set $\sigma_h^2 = 1$.

C. Comparing the distributions of $\bar{\lambda}_a$ and $\bar{\lambda}_c$. For both matrices, namely $\boldsymbol{A}_a$ and $\boldsymbol{A}_c$, we produced stochastic perturbations such that, at time t the demographic rates are affected following the procedure we outlined in the previous section. We then constructed the corresponding stochastic matrices $\boldsymbol{A}_{a,t}$ and $\boldsymbol{A}_{c,t}$. For simplicity here we refer to the stochastic matrices as $\boldsymbol{A}_t$, noting that the same procedure was followed for the full age-dependent and the constant-adult matrices. Thus, at each iteration we estimate the population vector $\boldsymbol{n}_t$ as

$$\boldsymbol{n}_t = \boldsymbol{A}_t \, \boldsymbol{n}_{t-1}, \qquad \text{[S11]}$$

where, at step t, we scale $\boldsymbol{n}_{t-1}$ to sum to unity as

$$\boldsymbol{w}_{t-1} = \frac{\boldsymbol{n}_{t-1}}{\sum_{x=0}^{\omega} n_{x,t-1}}, \qquad \text{[S12]}$$

and thus Eq. **S11** becomes $\boldsymbol{w}_t = \boldsymbol{A}_t \, \boldsymbol{w}_{t-1}$. After this standardization, we calculate the population growth rate at time t as

$$\lambda_t = \sum_{x=0}^{\omega} w_{x,t}. \qquad \text{[S13]}$$

We ran simulations of the perturbation analysis for $T = 200$ time steps, from which we calculated the averages

$$\bar{\lambda}_a = \frac{1}{T} \sum_{t=1}^{T} \lambda_{a,t}$$

and

$$\bar{\lambda}_c = \frac{1}{T} \sum_{t=1}^{T} \lambda_{c,t}.$$

We repeated these simulations 2 000 times for each combination of demographic rates and scenario. We then constructed the distributions of $\bar{\lambda}_a$ and $\bar{\lambda}_c$ and calculated Kullback-Leibler discrepancies (KL) (21, 26) between the resulting distributions. The KL discrepancies allowed us to determine the amount of information lost when estimating

the stochastic population growth rate with a one-adult-stage model. To calculate the KL values we defined $P_a(\bar{\lambda})$ as the distribution of $\bar{\lambda}_a$ for a given combination of mortality and fecundity trajectories and under one of the three scenarios, and $P_c(\bar{\lambda})$ the corresponding distribution for $\bar{\lambda}_c$. The Kullback-Leibler discrepancy of P_c with respect to P_a, namely $KL(P_a, P_c)$, is calculated as

$$KL(P_a, P_c) = \int_0^\infty P_a(\bar{\lambda}) \ln\left(\frac{P_a(\bar{\lambda})}{P_c(\bar{\lambda})}\right) d\bar{\lambda}. \qquad \textbf{[S14]}$$

Thus, if both distributions are equal, then $KL(P_a, P_c) = 0$ and there is therefore no loss of information. As $KL(P_a, P_c)$ increases, so does the loss of information.

To reduce the asymmetry between $KL(P_a, P_c)$ and $KL(P_c, P_a)$, and to make the results easier to interpret, we used McCulloch's (26) calibration. With this calibration, a $KL(P_a, P_c)$ is equal to to a KL value between a Bernouilli distribution with probability (parameter) 0.5 and one with parameter q. If q is close to 0.5, this means that P_a and P_c are as close to each other as $B(0.5)$ and $B(q)$. The value of q is obtained as

$$q(k) = \frac{1 + (1 - e^{-2k})^{\frac{1}{2}}}{2}, \qquad \textbf{[S15]}$$

where $k = KL(P_a, P_c)$. Thus, This calibration can be interpreted as a probability between 0.5 and 1, where 0.5 implies that both distributions are equal, and 1 that there is no overlap between them.

2. Review and alternative decomposition of E $[\lambda_t] = \lambda_e$ and Var $[\lambda_t] = V_\lambda$

The following section provides alternative decompositions for the expected value and variance of λ_t. However, these are not intended as new results but as calculations that can help us explain the results from our simulations. These alternative decompositions are concurrent with the extensive previous work on the estimation of the expected value of λ_t and approximations to the long-run stochastic population growth rate (12, 41, 44, 45), decompositions of the variance λ_t (6, 7, 13, 42), and on the effect of the covariation between demographic rates on this variance (4, 14, 42, 43).

A. Expressing λ_t in terms of demographic rates and age structure. We based our decompositions on the method proposed by Brown and Alexander (6) and Brown *et al.* (7), although as we mention above, we do not only focus on the variance of λ_t, but also on the expected value, which yields the expected population growth rate, λ_e. Furthermore, our method differs from theirs in that we decompose the variance in λ_t instead of the relative population growth rate δN_i, while we do not take into account several within year mortality and recruitment episodes but assume that all mortality and recruitment can be summarized by a single yearly rate for each age.

Under stochastic environments, the population growth rate is given by

$$\lambda_t = \frac{N_t}{N_{t-1}}, \qquad \textbf{[S16]}$$

where $N_t = \sum_{x=0}^{\omega} n_{x,t}$ is the total population size at time t and $n_{x,t}$ is the number of individuals in age x at time t, where ω is the maximum age the population can reach (42).

The equality in **S16** can be reformulated as

$$\lambda_t = \sum_{x=0}^{\omega} w_{x,t-1}(b_{x,t} + p_{x,t}), \qquad \textbf{[S17]}$$

where $b_x \geq 0$ is the age-specific fecundity rate, $p_x \in [0, 1]$ is the age-specific survival probability, while $w_{x,t-1} = n_{x,t-1}/N_{t-1}$ is the proportions of individuals in age x at time $t - 1$ (for a full derivation see (42)).

Thus, λ_t can be calculated as the sum of the proportion of individuals contributed by $n_{x,t-1}$ to the next time t. We will note this proportion as

$$y_{x,t} = w_{x,t-1}(b_{x,t} + p_{x,t}). \qquad \textbf{[S18]}$$

In addition, we define the expected population growth rate, noted as $\mathrm{E}[\lambda_t] = \lambda_e$, as the theoretical mean of λ_t. This expected value can be approximated as

$$\lambda_e \approx \bar{\lambda} = \frac{1}{t} \sum_{k=K}^{t} \lambda_k \qquad \textbf{[S19]}$$

for large t and where K is an initial burn-in period so that each simulated population reaches weak ergodicity in the age structure (11).

Our derivations below also require to define the expected values for the demographic rates as $\mathrm{E}[b_{x,t}] = \beta_x$ and $\mathrm{E}[p_{x,t}] = \rho_x$ for all $x \in \Omega$, where $\Omega = \{x \in \mathbb{N}_0 \mid x \leq \omega\}$ is the set of discrete ages from 0 to the maximum age ω. These are the expected values of the age-specific fecundity rates and survival probabilities, respectively, while $\mathrm{E}[w_{x,t}] = \eta_x$ is the expected values of the proportion of individuals in age x.

B. Expected value of λ_t. Here we provide a decomposition of λ_e based on the definition of λ_t in Eq. **S17**.

Let $b_{x,t} \geq 0$ and $p_{x,t} \in [0,1]$ be the age- and time-specific fecundity rate and survival probability for age x at time t of a given population, and let $w_{x,t-1} \in [0,1]$ be the proportion of individuals in age x at time $t-1$, for $x = 0, 1, 2, \ldots, \omega$ and $t > 0$, where ω is the maximum age the population can reach. If $b_{x,t}$, $p_{x,t}$ and $w_{x,t-1}$, are age- and time-specific random variables with expected values given by $\mathrm{E}[b_{x,t}] = \beta_x$, $\mathrm{E}[p_{x,t}] = \rho_x$, and $\mathrm{E}[w_{x,t-1}] = \eta_x$, then the expected population growth rate, $\mathrm{E}[\lambda_t] = \lambda_e$, will be

$$\lambda_e = \tilde{\lambda} + C_{wb} + C_{wp}, \quad \textbf{[S20]}$$

where

$$\tilde{\lambda} = \sum_{x=0}^{\omega} \eta_x \left(\beta_x + \rho_x\right),$$

$$C_{wb} = \sum_{x=0}^{\omega} \mathrm{Cov}\left[w_{x,t-1}, b_{x,t}\right]$$

and

$$C_{wp} = \sum_{x=0}^{\omega} \mathrm{Cov}\left[w_{x,t-1}, p_{x,t}\right].$$

Proof. Based on the results in Eqs. **S17** and **S18**, we have that the expected value of λ_t can be expressed as the expectation of the sum of the elements in the vector of proportions $\boldsymbol{y}_t^\top = [y_{0,t}, y_{1,t}, \ldots, y_{\omega,t}]$, such that

$$\begin{aligned}
\mathrm{E}[\lambda_t] &= \mathrm{E}\left[\sum_{x=0}^{\omega} y_{x,t}\right] \\
&= \mathrm{E}\left[\sum_{x=0}^{\omega} w_{x,t-1}(b_{x,t} + p_{x,t})\right] \\
&= \sum_{x=0}^{\omega} \mathrm{E}\left[w_{x,t-1}(b_{x,t} + p_{x,t})\right] \\
&= \sum_{x=0}^{\omega} \left(\mathrm{E}[w_{x,t-1} b_{x,t}] + \mathrm{E}[w_{x,t-1} p_{x,t}]\right).
\end{aligned}$$

Since by definition the covariance between any pair of random variables X and Y is $\mathrm{Cov}[X,Y] = \mathrm{E}[XY] - \mathrm{E}[X]\,\mathrm{E}[Y]$, then it follows that the expected value of their product will be $\mathrm{E}[XY] = \mathrm{Cov}[X,Y] + \mathrm{E}[X]\,\mathrm{E}[Y]$. Based on this definition, we can express the expected value of the yearly population growth rate as

$$\begin{aligned}
\mathrm{E}[\lambda_t] = \sum_{x=0}^{\omega} \big(&\mathrm{Cov}[w_{x,t-1}, b_{x,t}] + \mathrm{E}[w_{x,t-1}]\,\mathrm{E}[b_{x,t}] \\
&+ \mathrm{Cov}[w_{x,t-1}, p_x] + \mathrm{E}[w_{x,t-1}]\,\mathrm{E}[p_{x,t}]\big),
\end{aligned}$$

which can be rearranged as

$$\begin{aligned}
\mathrm{E}[\lambda_t] &= \sum_{x=0}^{\omega} \mathrm{Cov}[w_{x,t-1}, b_{x,t}] + \sum_{x=0}^{\omega} \mathrm{Cov}[w_{x,t-1}, p_{x,t}] \\
&\quad + \sum_{x=0}^{\omega} \mathrm{E}[w_{x,t-1}] (\mathrm{E}[b_{x,t}] + \mathrm{E}[p_{x,t}]) \\
&= \sum_{x=0}^{\omega} \mathrm{Cov}[w_{x,t-1}, b_{x,t}] + \sum_{x=0}^{\omega} \mathrm{Cov}[w_{x,t-1}, p_{x,t}] \\
&\quad + \sum_{x=0}^{\omega} \eta_x (\beta_x + \rho_x).
\end{aligned}$$

□

However, under serially independent environments we have that $C_{wb} = C_{wp} = 0$, which simplifies equation **S20** to

$$\lambda_e = \tilde{\lambda}. \qquad \textbf{[S21]}$$

C. Variance of λ_t. Following the initial statement as in the previous section, whereby if $b_{x,t}$, $p_{x,t}$ and $w_{x,t-1}$ are age- and time-dependent random variables for the fecundity rates, survival probabilities, and proportion of individuals in age $x \in \Omega$, respectively, then the variance of the stochastic population growth rate, $\mathrm{Var}[\lambda_t] = V_\lambda$, is

$$V_\lambda = V_{wb} + V_{wp} + 2C_x + 2C_{ij}, \qquad \textbf{[S22]}$$

where

$$V_{wb} = \sum_{x=0}^{\omega} \mathrm{Var}[w_{x,t-1} b_{x,t}],$$

$$V_{wp} = \sum_{x=0}^{\omega} \mathrm{Var}[w_{x,t-1} p_{x,t}],$$

$$C_x = \sum_{x=0}^{\omega} \mathrm{Cov}[w_{x,t-1} b_{x,t}, w_{x,t-1} p_{x,t}],$$

and

$$\begin{aligned}
C_{ij} &= \sum_{0 \le i < j \le \omega} \{\mathrm{Cov}[w_{i,t-1} b_{i,t}, w_{j,t-1} b_{j,t}] + \mathrm{Cov}[w_{i,t-1} p_{i,t}, w_{j,t-1} p_{j,t}] \\
&\quad + \mathrm{Cov}[w_{i,t-1} b_{i,t}, w_{j,t-1} p_{j,t}] + \mathrm{Cov}[w_{i,t-1} p_{i,t}, w_{j,t-1} b_{j,t}]\}
\end{aligned}$$

Proof. Based on the results in Eqs. **S17** and **S18**, we have that

$$\mathrm{Var}[\lambda_t] = \mathrm{Var}\left[\sum_{x=0}^{\omega} y_{x,t}\right].$$

By definition the variance of the sum of n random variables $X_1, X_2, \ldots, X_n$ is

$$\mathrm{Var}\left[\sum_{i=1}^{n} X_i\right] = \sum_{i=1}^{n} \mathrm{Var}[X_i] + 2 \sum_{0 \le i < j < n} \mathrm{Cov}[X_i, X_j].$$

Thus, following this definition we can express the variance of λ_t as

$$\begin{aligned}
\mathrm{Var}[\lambda_t] &= \mathrm{Var}\left[\sum_{x=0}^{\omega} y_{x,t}\right] \\
&= \sum_{x=0}^{\omega} \mathrm{Var}[y_{x,t}] + 2 \sum_{0 \le i < j \le \omega} \mathrm{Cov}[y_{i,t}, y_{j,t}],
\end{aligned} \qquad \textbf{[S23]}$$

Therefore, to calculate the variance in λ_t it is necessary first to estimate the variance for the elements of the vector of proportions $\boldsymbol{y}_t^\top = [y_{0,t}, y_{1,t}, \ldots, y_{\omega,t}]$. For simplicity of notation, we will not include the subindices t and $t-1$. Thus, based on Eq. **S18**, we have that

$$\mathrm{Var}\left[y_x\right] = \mathrm{Var}\left[w_x(b_x + p_x)\right] \quad \text{for } x \in \Omega.$$

Expressing the variance in terms of its expectation and using the property $\mathrm{Var}\left[X\right] = \mathrm{E}\left[X^2\right] - \mathrm{E}\left[X\right]^2$ for any random varialbe X, we obtain

$$\begin{aligned} \mathrm{Var}\left[y_x\right] &= \mathrm{E}\left[(w_x b_x + w_x p_x)^2\right] - \mathrm{E}\left[w_x b_x + w_x p_x\right]^2 \\ &= \mathrm{E}\left[(w_x b_x + w_x p_x)^2\right] - \mathrm{E}\left[w_x b_x + w_x p_x\right] \mathrm{E}\left[w_x b_x + w_x p_x\right]. \end{aligned}$$

Expanding the square we have that

$$\begin{aligned} \mathrm{Var}\left[y_x\right] &= \mathrm{E}\left[w_x^2 b_x^2 + 2 w_x^2 b_x p_x + w_x^2 p_x^2\right] \\ &\quad - \mathrm{E}\left[w_x b_x\right]^2 - 2\mathrm{E}\left[w_x b_x\right] \mathrm{E}\left[w_x p_x\right] - \mathrm{E}\left[w_x p_x\right]^2 \\ &= \mathrm{E}\left[w_x^2 b_x^2\right] + 2\,\mathrm{E}\left[w_x^2 b_x p_x\right] + \mathrm{E}\left[vx^2 p_x^2\right] \\ &\quad - \mathrm{E}\left[w_x b_x\right]^2 - 2\mathrm{E}\left[w_x b_x\right] \mathrm{E}\left[w_x p_x\right] - \mathrm{E}\left[w_x p_x\right]^2 \\ &= \mathrm{Var}\left[w_x b_x\right] + \mathrm{Var}\left[w_x p_x\right] + 2\,\mathrm{Cov}\left[w_x b_x, w_x p_x\right]. \end{aligned}$$

Including the corresponding sub-indexes we have that the equation above becomes

$$\begin{aligned} \mathrm{Var}\left[y_{x,t}\right] &= \mathrm{Var}\left[w_{x,t-1} b_{x,t}\right] + \mathrm{Var}\left[w_{x,t-1} p_{x,t}\right] \\ &\quad + 2\,\mathrm{Cov}\left[w_{x,t-1} b_{x,t}, w_{x,t-1} p_{x,t}\right]. \quad \text{for } x \in \Omega. \end{aligned} \qquad \textbf{[S24]}$$

Next, we require providing the covariance between any two elements $y_{i,t}$ and $y_{j,t}$ of $\boldsymbol{y}$, where $i \neq j$ and $i, j \in \Omega$. Once more, we will still omit the subindices t and $t-1$. First, we can express the covariance between any pair of porportions y_i and y_j in terms of expectations as

$$\mathrm{Cov}\left[y_i, y_j\right] = \mathrm{E}\left[y_i y_j\right] - \mathrm{E}\left[y_i\right] \mathrm{E}\left[y_j\right], \quad \forall i, j \in \Omega \text{ and } i \neq j.$$

Then, we can replace $y_{i,t}$ and $y_{j,t}$ by their corresponding functions of demographic rates as in Eq. **S18** which yields

$$\begin{aligned} \mathrm{Cov}\left[y_i, y_j\right] &= \mathrm{E}\left[w_i(b_i + p_i)\, w_j(b_j + p_j)\right] \\ &\quad - \mathrm{E}\left[w_i(b_i + p_i)\right] \mathrm{E}\left[w_j(b_j + p_j)\right]. \end{aligned}$$

Expanding the products we have that

$$\begin{aligned} \mathrm{Cov}\left[y_i, y_j\right] &= \mathrm{E}\left[w_i b_i w_j b_j + w_i b_i w_j p_j + w_j b_j w_i p_i + w_i p_i w_j p_j\right] \\ &\quad - \left(\mathrm{E}\left[w_i b_i\right] + \mathrm{E}\left[w_i p_i\right]\right)\left(\mathrm{E}\left[w_j b_j\right] + \mathrm{E}\left[w_j p_j\right]\right)\}. \end{aligned}$$

Next, based on the linearity property of the expected value we have that

$$\begin{aligned} \mathrm{Cov}\left[y_{i,t}, y_{j,t}\right] &= \mathrm{E}\left[w_i b_i\; w_j b_j\right] + \mathrm{E}\left[w_i b_i\; w_j p_j\right] + \mathrm{E}\left[w_j b_j\; w_i p_i\right] + \mathrm{E}\left[w_i p_i\; w_j p_j\right] \\ &\quad - \mathrm{E}\left[w_i b_i\right] \mathrm{E}\left[w_j b_j\right] - \mathrm{E}\left[w_i p_i\right] \mathrm{E}\left[w_j b_j\right] \\ &\quad - \mathrm{E}\left[w_j p_j\right] \mathrm{E}\left[w_i b_i\right] - \mathrm{E}\left[w_i p_i\right] \mathrm{E}\left[w_j p_j\right], \end{aligned}$$

which, rearranging terms allows us to express it as sums of covariances between demographic rates as

$$\begin{aligned} \mathrm{Cov}\left[y_i, y_j\right] &= \mathrm{Cov}\left[w_i b_i, w_j b_j\right] + \mathrm{Cov}\left[w_i p_i, w_j p_j\right] \\ &\quad + \mathrm{Cov}\left[w_i b_i, w_j p_j\right] + \mathrm{Cov}\left[w_i p_i, w_j b_j\right]. \end{aligned}$$

Including the subindex for time, the equation above becomes

$$\begin{aligned} \mathrm{Cov}\left[y_{i,t}, y_{j,t}\right] &= \mathrm{Cov}\left[w_{i,t-1} b_{i,t}, w_{j,t-1} b_{j,t}\right] + \mathrm{Cov}\left[w_{i,t-1} p_{i,t}, w_{j,t-1} p_{j,t}\right] \\ &\quad + \mathrm{Cov}\left[w_{i,t-1} b_{i,t}, w_{j,t-1} p_{j,t}\right] \\ &\quad + \mathrm{Cov}\left[w_{i,t-1} p_{i,t}, w_{j,t-1} b_{j,t}\right], \quad \forall i \neq j \text{ and } i, j \in \Omega. \end{aligned} \qquad \textbf{[S25]}$$

Based on the results in Eqs. **S23**, **S24**, and **S25**, the variance of λ_t can then be calculated as

$$\begin{aligned}\mathrm{Var}\left[\lambda_t\right] &= \sum_{x=0}^{\omega}\left\{\mathrm{Var}\left[w_{x,t-1}b_{x,t}\right]+\mathrm{Var}\left[w_{x,t-1}p_{x,t}\right]+2\,\mathrm{Cov}\left[w_{x,t-1}b_{x,t},w_{x,t-1}p_{x,t}\right]\right\}\\ &+2\sum_{0\le i<j\le\omega}\left\{\mathrm{Cov}\left[w_{i,t-1}b_{i,t},w_{j,t-1}b_{j,t}\right]+\mathrm{Cov}\left[w_{i,t-1}p_{i,t},w_{j,t-1}p_{j,t}\right]\right.\\ &\left.+\mathrm{Cov}\left[w_{i,t-1}b_{i,t},w_{j,t-1}p_{j,t}\right]+\mathrm{Cov}\left[w_{i,t-1}p_{i,t},w_{j,t-1}b_{j,t}\right]\right\}.\end{aligned} \quad \textbf{[S26]}$$

□

3. Approximations to E $[r_t] = r_e$

For any random variable X, with mean $\mathrm{E}[X] = \mu_X$ and variance $\mathrm{E}\left[(X-\mu_x)^2\right] = \sigma_X^2$, and a function $f(X)$ $n \geq 2$ times differentiable in the range of X, we have that the expectation $\mathrm{E}[f(X)] = r_e$, known as the long-run population growth rate (45), can be approximated with the second order Taylor expansion given by

$$\begin{aligned}\mathrm{E}\left[f(X)\right] &\approx \mathrm{E}\left[f(\mu_X)+f'(\mu_X)(X-\mu_X)+\frac{1}{2}f''(\mu_X)(X-\mu_X)^2\right]\\ &= f(\mu_X)+f'(\mu_X)+\mathrm{E}\left[X-\mu_X\right]+\frac{1}{2}f''(\mu_X)\mathrm{E}\left[(X-\mu_X)^2\right]\\ &= f(\mu_X)+\frac{1}{2}f''(\mu_X)\sigma_X^2.\end{aligned}$$

Thus, for $f(\lambda_t) = \ln\lambda_t = r_t$, where $\mathrm{E}[\lambda_t] = \lambda_e$ calculated as in Eq. **S20** and $\mathrm{Var}[\lambda_t] = V_\lambda$ from Eq. **S22**, the second order Taylor approximation to $\mathrm{E}[r_t] = r_e$ will take the form

$$r_e \approx \ln(\lambda_e) - \frac{1}{2\lambda_e^2}V_\lambda. \quad \textbf{[S27]}$$

This formulation is concurrent with the small noise approximation to the long-run population growth rate proposed by Tuljapurkar (45) for serially uncorrelated environments, given by

$$\ln\lambda_s \approx \ln\lambda_0 - \frac{\tau_0^2}{2\lambda_0^2}, \quad \textbf{[S28]}$$

where λ_0 is the dominant eigenvector of the matrix of average rates, and matrix τ_0^2 accounts for the variances of and covariances between the average demographic rates scaled by the sensitivities of λ_0 to them. In other words, τ_0^2 provides an approximation to the variance in the population growth rate. The difference between Eqs. **S27** and **S28** is that the first is based on λ_e, which requires the estimation of the average demographic rates and average age-structure, while the second only depends on the matrix of average demographic rates. Both require averaging over the full environmental series. Thus, we would like to stress that the approximation in Eq. **S27** requires long-term stochastic simulations and, therefore, cannot be based on a deterministic calculation, while Tuljapurkar and Haridas (44) provided a simplified method to calculate the small-noise approximation in Eq. **S28**.

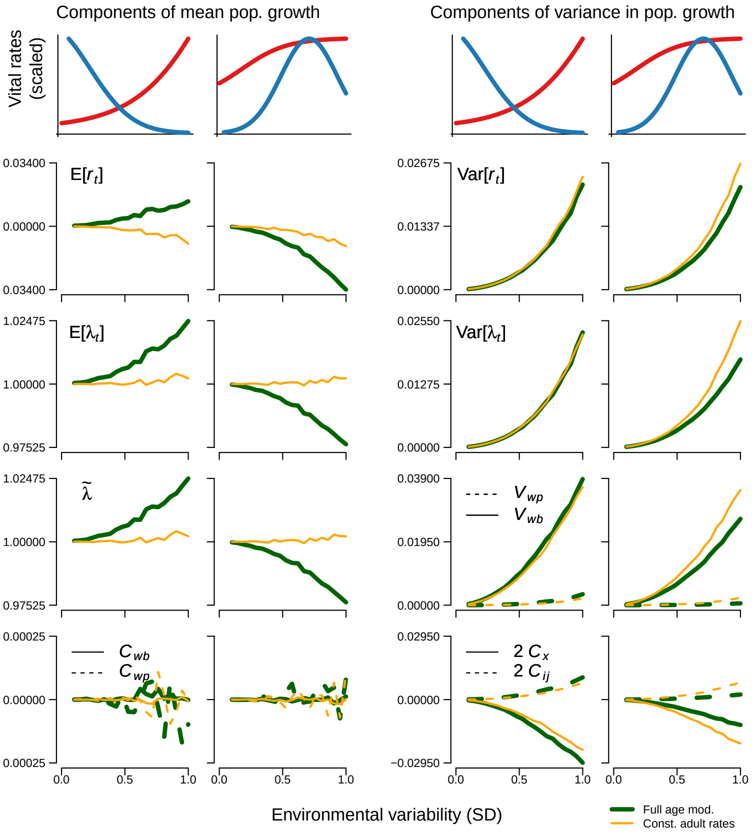

Fig. S1. Relationship between environmental variability (measured as the standard deviation of environmental shocks) and the components of E $[\lambda_t] = \lambda_e$ and Var $[\lambda_t] = V_\lambda$ for two combinations of mortality and fecundity profiles (F1-M3 and F4-M4 in Fig. 3) under the no covariation scenario (i.e. survival and fecundity are independent). The dark green thick lines correspond to the fully age-dependent models and the orange thin lines to the model with a single adult-stage.

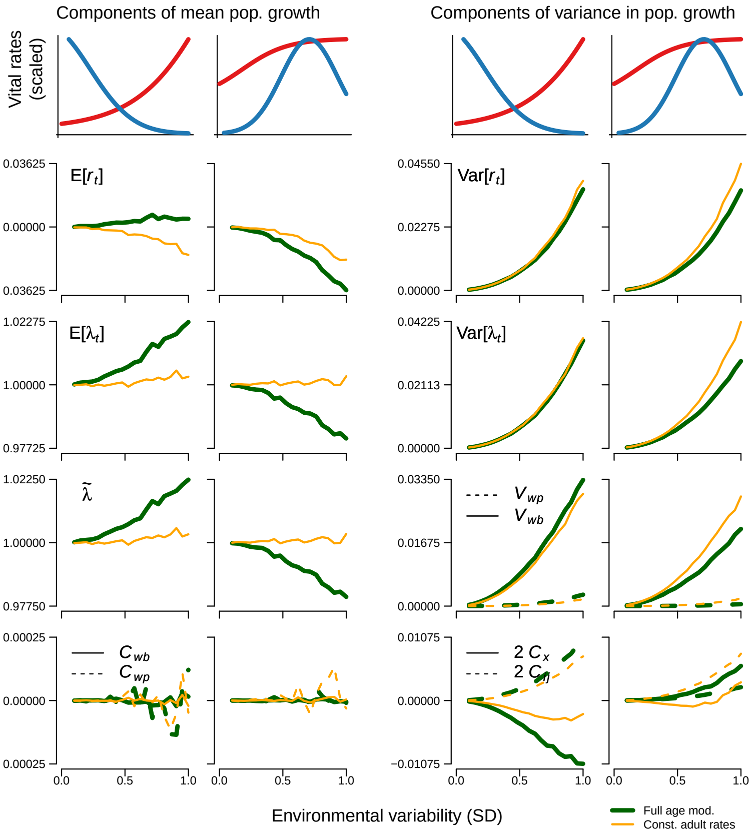

Fig. S2. Relationship between environmental variability (measured as the standard deviation of environmental shocks) and the components of E $[\lambda_t] = \lambda_e$ and Var $[\lambda_t] = V_\lambda$ for two combinations of mortality and fecundity profiles (F1-M3 and F4-M4 in Fig. 3) under the positive covariation scenario (i.e. survival and fecundity vary in the same direction and magnitude). The dark green thick lines correspond to the fully age-dependent models and the orange thin lines to the model with a single adult-stage.

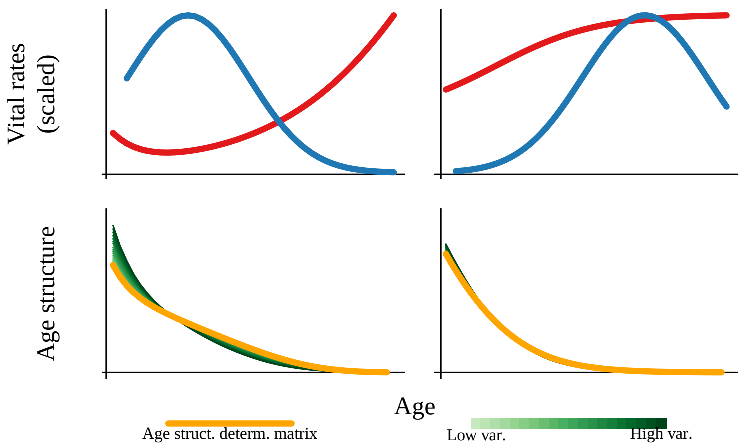

Fig. S3. Change in the mean age structure as a function of increasing environmental variability for two combinations of mortality and fecundity.

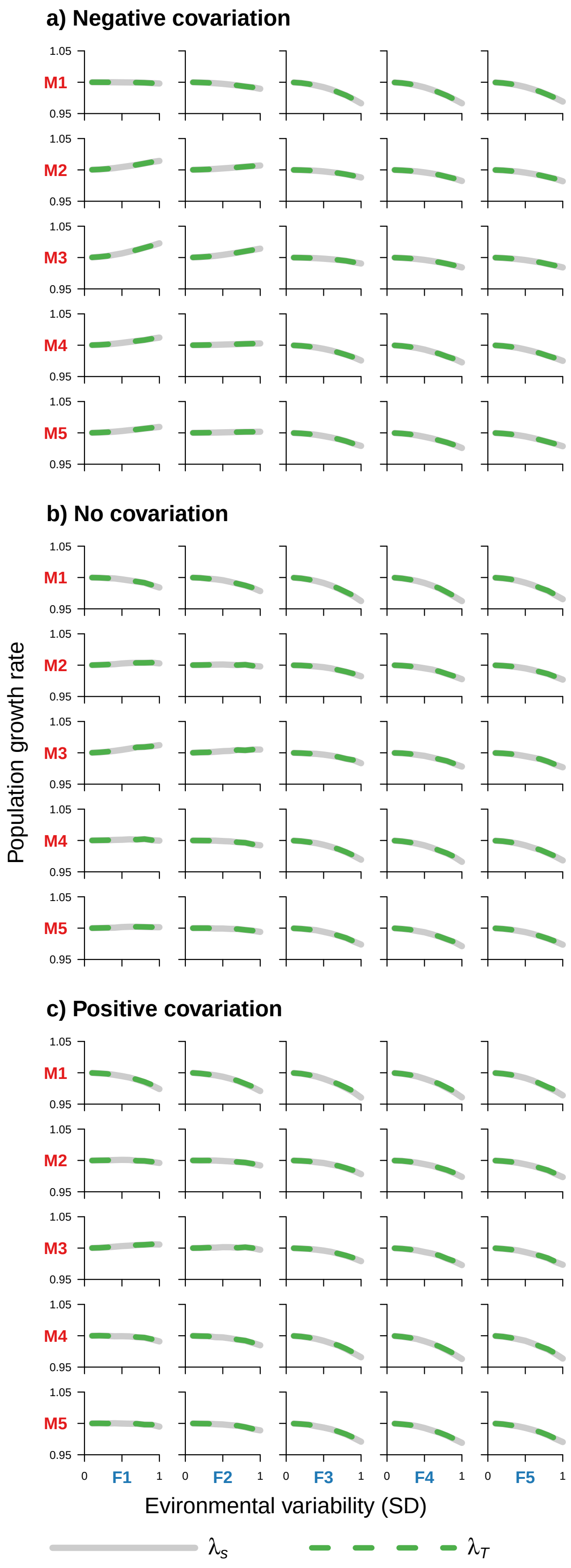

Fig. S4. The long-run population growth rate, λ_s, under increasing environmental variation, and the first order approximation, λ_T, based on λ_e and V_λ as in Eq. (6) in the main text. The combinations of mortality (M1-M5) and of fecundity (F1-F5) are distributed as in Fig. 3.

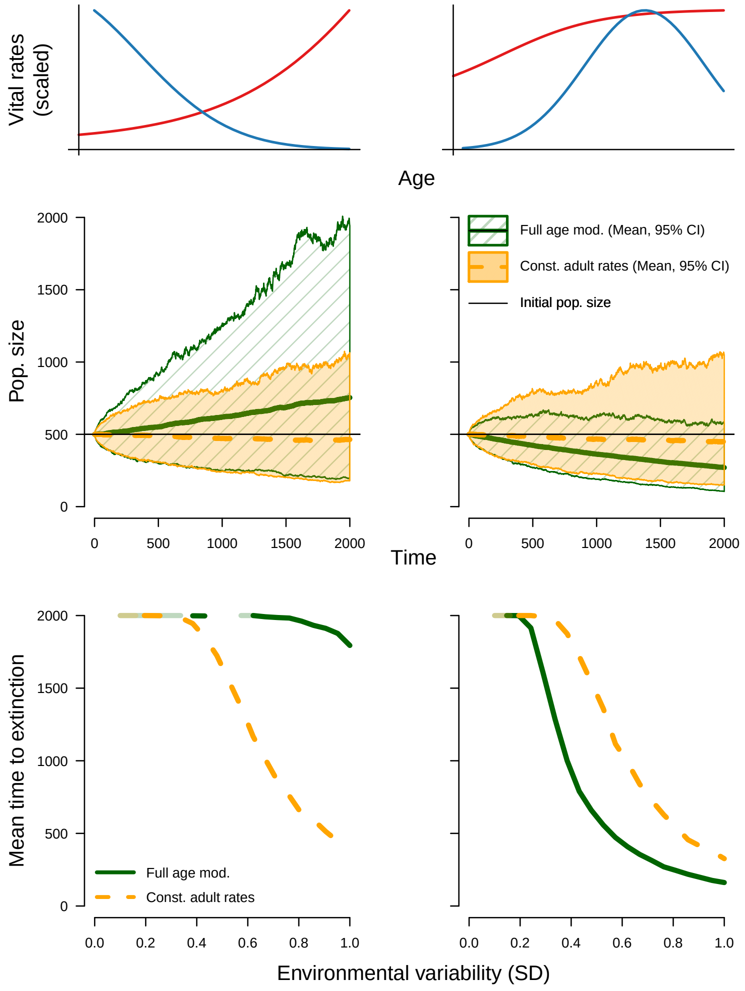

Fig. S5. Population sizes after 2,000 time steps and mean time to extinction for two combinations of mortality and fecundity (F1-M3 and F4-M4 in Fig. 3) under the no covariation scenario (i.e. survival and fecundity are independent). The lightly shaded lines in the lower panels indicate that no populations went extinct.

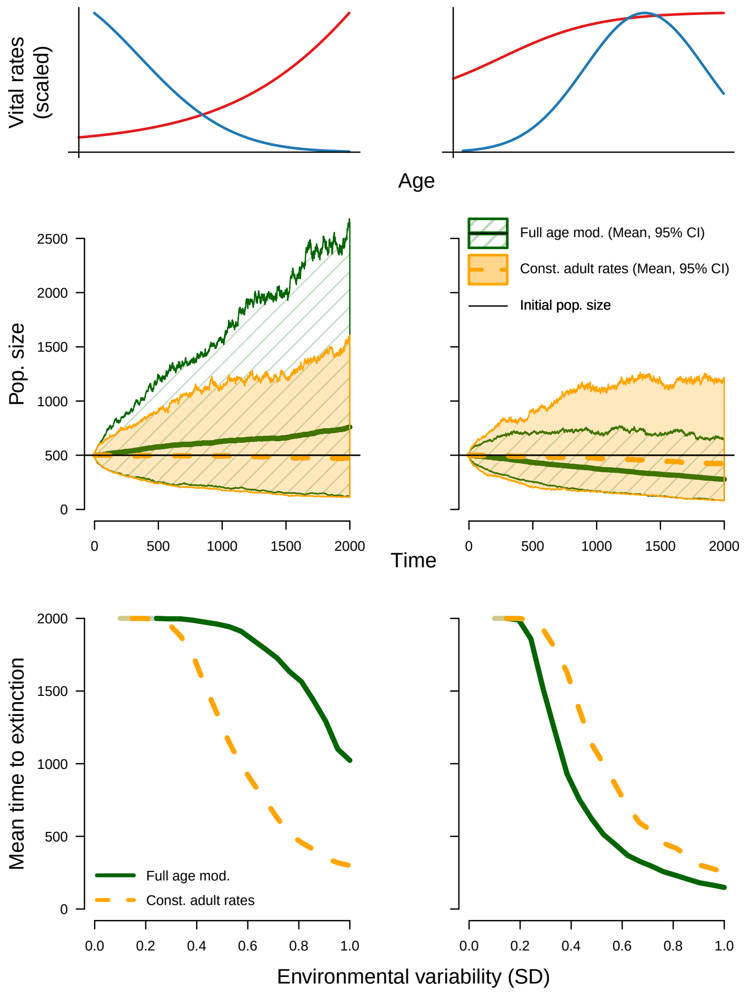

Fig. S6. Population sizes after 2,000 time steps and mean time to extinction for two combinations of mortality and fecundity (F1-M3 and F4-M4 in Fig. 3) under the positive covariation scenario (i.e. survival and fecundity vary in the same direction and magnitude). The lightly shaded lines in the lower panels indicate that no populations went extinct.

Table S1. Description of the datasets used on the Bayesian survival trajectory analysis (BaSTA) to estimate age-specific mortality

Common name	Species	Class	Num. Obs.	Age matur.	Study	refs
Lion	*Panthera leo*	Mammal	500	3 years	1966-2010	(28)
European badger	*Meles meles*	Mammal	1103	1 years	1984-2005	(19)
Savannah baboon	*Papio cynocephalus*	Mammal	1030	4 years	1970-2008	(1)
Azara's owl monkey	*Aotus azarae*	Mammal	240	2 years	1992-2012	(16)
Golden-headed lion tamarin	*Leontopithecus chrysomelas*	Mammal	245	2 years	1992-2006	(49)
Red deer	*Cervus elaphus*	Mammal	2829	2 years	1974-2011	(9)
Soay sheep	*Ovis aries*	Mammal	2549	2 years	1995-2005	(10)
Roe deer	*Capreolus capreolus*	Mammal	493	1 years	1985-2012	(22)
New Zealand long-tailed bat	*Chalinolobus tuberculatus*	Mammal	2853	2 years	1993-2012	(32, 33)
Wood mouse	*Apodemus sylvaticus*	Mammal	264	2 months	1000-1033	(25)
Sparrowhawk	*Accipiter nisus*	Bird	714	1 years	1971-1997	(29)
Kestrel	*Falco tinnunculus*	Bird	878	1 years	1974-1989	(48)
Southern fulmar	*Fulmarus glacialoides*	Bird	1189	7 years	1964-2011	(20)
Common tern	*Sterna hirundo*	Bird	1763	3 years	1992-2012	(37, 40)
Rook	*Corvus frugilegus*	Bird	935	2 years	1964-1973	(30)
Great tit	*Parus major*	Bird	4935	1 years	1960-2006	(3)
Asp viper	*Vipera aspis*	Reptile	161	3 years	1986-2010	(2)
Garter snake	*Thamnophis elegans*	Reptile	1652	4 years	1980-1996	(5, 27)
Smooth snake	*Coronella austriaca*	Reptile	203	6 years	1992-2012	(35)
Spotted skink	*Niveoscincus ocellatus*	Reptile	616	2 years	2000-2011	(46)
Common European toad	*Bufo bufo*	Amphibian	5692	0 years	1993-2004	(36)
European green toad	*Pseudepidalea viridis*	Amphibian	1207	0 years	1996-2005	–
Red-spotted newt	*Notophthalmus viridescens*	Amphibian	1079	0 years	1975-1983	(17)
Fire salamander	*Salamandra salamandra*	Amphibian	535	0 years	1965-1982	(38)

References

1. Alberts, S., Hollister-Smith, J., Mututua, R., Sayialel, S., Muruthi, P., Warutere, J. & Altmann, J. (2005). *Seasonality in Primates: Studies of Living and Extinct Human and Non-Human Primates*, Cambridge University Press, chap. Seasonality and long-term change in a savannah environment, pp. 157–196.
2. Altwegg, R., Dummermuth, S., Anholt, B.R. & Flatt, T. (2005). Winter weather affects asp viper (*Vipera aspis aspis*) population dynamics through susceptible juveniles. *Oikos*, 110, 55–66.
3. Bouwhuis, S., Choquet, R., Sheldon, B.C. & Verhulst, S. (2012). The form and fitness cost of senescence: age-specific recapture, survival, reproduction and reproductive value in a wild bird population. *American Naturalist*, 179, E15–E27.
4. Boyce, M.S., Haridas, C. & Lee, C. (2006). Demography in an increasingly variable world. *Trends in Ecology & Evolution*, 21, 141–148.
5. Bronikowski, A.M. & Arnold, S.J. (1999). The evolutionary ecology of life history variation in the garter snake *Thamnophis elegans*. *Ecology*, 80, 2314–2325.
6. Brown, D. & Alexander, N. (1991). The Analysis of the Variance and Covariance of Products. *Biometrics*, 47, 429.
7. Brown, D., Alexander, N., Marrs, R.W. & Albon, S. (1993). Structured accounting of the variance of demographic change. *Journal of Animal Ecology*, 62, 490.
8. Caswell, H. (2001). *Matrix Population Models: Construction, Analysis and Interpretation*. Second edition edn. Sinauer Associates, Sunderland, MA.
9. Clutton-Brock, T.H., Guinness, F.E. & Albon, S.D. (1982). *Red deer: the behaviour and ecology of two sexes*. University of Chicago Press.
10. Clutton-Brock, T.H. & Pemberton, J.M. (2004). *Soay Sheep: Dynamics and Selection in an Island Population*. Cambridge University Press, Cambridge.
11. Cohen, J.E. (1976). Ergodicity of Age Structure in Populations with Markovian Vital Rates, I: Countable States. *Journal Of The American Statistical Association*, 71, 335–339.
12. Cohen, J.E. (1979). Long-run growth rates of discrete multiplicative processes in Markovian environments. *Journal of Mathematical Analysis and Applications*, 69, 243–251.
13. Coulson, T., Gaillard, J. & Festa-Bianchet, M. (2005). Decomposing the variation in population growth into contributions from multiple demographic rates. *Journal of Animal Ecology*, 74, 789–801.
14. Doak, D.F., Morris, W.F., Pfister, C., Kendall, B.E. & Bruna, E.M. (2005). Correctly estimating how environmental stochasticity influences fitness and population growth. *The American Naturalist*, 166, E14–E21.
15. Engen, S., Lande, R., Saether, B.E. & Weimerskirch, H. (2005). Extinction in relation to demographic and environmental stochasticity in age-structured models. *Mathematical Biosciences*, 195, 210–227.
16. Fernandez-Duque, E. & Huck, M. (2013). Till death (or an intruder) do us part: intra-sexual competition in a monogamous primate. *PLoS One*, 8, e53724.
17. Gill, D.E. (1985). Interpreting breeding patterns from census data: A solution to the husting dilemma. *Ecology*, 66, 344Ð354.
18. Gompertz, B. (1825). On the nature of the function expressive of the law of human mortality, and on a new mode of determining the value of life contingencies. *Philosophical Transactions of the Royal Society of London*, 115, 513–583.
19. Graham, J., Smith, G.C., Delahay, R.J., Bailey, T., McDonald, R.A. & Hodgson, D. (2013). Multi-state modelling reveals sex-dependent transmission, progression and severity of tuberculosis in wild badgers. *Epidemiology and Infection*, 141, 1429–1436.
20. Jenouvrier, S., Barbraud, C. & Weimerskirch, H. (2003). Effects of climate variability on the temporal population dynamics of southern fulmars. *Journal of Animal Ecology*, 72, 576–587.
21. Kullback, S. & Leibler, R.A. (1951). On information and sufficiency. *The Annals of Mathematical Statistics*, 22, 79–86.
22. Lemaître, J.F. & Gaillard, J.M. (2013). Male survival patterns do not depend on male allocation to sexual competition in large herbivores. *Behavioral Ecology*, 24, 421–428.
23. Leslie, P. (1945). On the Use of Matrices in Certain Population Mathematics. *Biometrika*, 33, 183–212.
24. Makeham, W.M. (1866). On the law of mortality. *Journal of the Institute of Actuaries*, 13, 1–34.
25. Malo, A.F., Godsall, B., Prebble, C., Grange, Z., McCandless, S., Taylor, A. & Coulson, T. (2013). Positive effects of an invasive shrub on aggregation and abundance of a native small rodent. *Behavioral Ecology*, 24, 759–767.
26. McCulloch, R. (1989). Local model influence. *Journal Of The American Statistical Association*, 84, 473–478.
27. Miller, D.A., Clark, W.R., Arnold, S.J. & Bronikowski, A.M. (2011). Stochastic population dynamics in

populations of western terrestrial garter snakes with divergent life histories. *Ecology*, 92, 1658–1671.
28. Mosser, A., Fryxell, J., Eberly, L. & Packer., C. (2009). Serengeti real estate: density versus fitness-based indicators of lion habitat quality. *Ecology Letters*, 12, 1050–1060.
29. Newton, I. & Rothery, P. (1997). Senescence and reproductive value in sparrowhawks. *Ecology*, 78, 1000–1008.
30. Patterson, I.J., Dunnet, G.M. & Goodbody, S.R. (1988). Body weight and juvenile mortality in rooks corvus frugilegus. *Journal of Animal Ecology*, 57, 1041Ð1052.
31. Pletcher, S. (1999). Model fitting and hypothesis testing for age-specific mortality data. *Journal of Evolutionary Biology*, 12, 430–439.
32. Pryde, M.A., Lettink, M. & O'Donnell, C.F.J. (2006). Survivorship in two populations of long-tailed bats (*Chalinolobus tuberculatus*) in new zealand. *New Zealand Journal of Zoology*, 33, 85–95.
33. Pryde, M.A., O'Donnell, C.F.J. & Barker, R.J. (2005). Factors inßuencing survival and long-term population viability of new zealand long-tailed bats (chalinolobus tuberculatus): Implications for conservation. *Biological Conservation*, 126, 175–185.
34. R Core Team (2018). *R: A Language and Environment for Statistical Computing*. R Foundation for Statistical Computing, Vienna, Austria.
35. Reading, C.J. (2004). Age, growth and sex determination in a population of smooth snakes, coronella austriaca, in southern england. *Amphibia-Reptilia*, 25, 137– 150.
36. Reading, C.J. (2007). Linking global warming to amphibian declines through its effects on female body condition and survivorship. *Oecologia*, 151, 125–131.
37. Rebke, M., Coulson, T., Becker, P. & Vaupel, J.W. (2010). Reproductive improvement and senescence in a long-lived bird. *Proceedings of the National Academy of Sciences of the United States of America*, 107, 7841–7846.
38. Schmidt, B.R., Feldmann, R. & Schaub, M. (2005). Demographic processes underlying population growth and decline in *Salamandra salamandra*. *Conservation Biology*, 19, 1149Ð1156.
39. Siler, W. (1979). A competing-risk model for animal mortality. *Ecology*, 60, 750–757.
40. Szostek, L. & Becker, P.H. (2012). Terns in trouble: Demographic consequences of low breeding success and recruitment on a common tern population in the german wadden sea. *Journal of Ornithology*, 153, 313–326.
41. Tuljapurkar, S. (1989). An uncertain life: demography in random environments. *Theoretical Population Biology*, 35, 227–294.
42. Tuljapurkar, S. (1990). *Population Dynamics in Variable Environments*. Springer-Verlag, Berlin.
43. Tuljapurkar, S., Gaillard, J.M. & Coulson, T. (2009). From stochastic environments to life histories and back. *Philosophical transactions of the Royal Society of London Series B, Biological sciences*, 364, 1499–1509.
44. Tuljapurkar, S. & Haridas, C.V. (2006). Temporal autocorrelation and stochastic population growth. *Ecology Letters*, 9, 327–337.
45. Tuljapurkar, S.D. (1982). Population dynamics in variable environments. II. Correlated environments, sensitivity analysis and dynamics. *Theoretical Population Biology*, 21, 114–140.
46. Uller, T., While, G.M., Cadby, C.D., Harts, A., OÕConnor, K., Pen, I. & Wapstra, E. (2011). Thermal opportunity, maternal effects, and offspring survival at different climatic extremes in a viviparous lizard. *Evolution*, 65, 2313Ð2324.
47. Vaupel, J.W., Baudisch, A., Dölling, M., Roach, D.A. & Gampe, J. (2004). The case for negative senescence. *Theoretical Population Biology*, 65, 339–351.
48. Village, A. (1990). *The Kestrel*. T & AD Poyser Ltd, London.
49. Zeigler, S.L., Vleeschouwer, K.M.D. & Raboy, B.E. (2013). Assessing extinction risk in small metapopulations of golden-headed lion tamarins (*Leontopithecus chrysomelas*) in bahia state, brazil. *Biotropica*, 45, 528–535.
